# Fabrication of Mediatorless/Membraneless Glucose/Oxygen Based Biofuel Cell using Biocatalysts Including Glucose Oxidase and Laccase Enzymes

**DOI:** 10.1038/srep30128

**Published:** 2016-07-18

**Authors:** Marcelinus Christwardana, Ki Jae Kim, Yongchai Kwon

**Affiliations:** 1 Graduate school of Energy and Environment, Seoul National University of Science and Technology, 232 Gongneung-ro, Nowon-gu, Seoul, 01811, Republic of Korea

## Abstract

Mediatorless and membraneless enzymatic biofuel cells (EBCs) employing new catalytic structure are fabricated. Regarding anodic catalyst, structure consisting of glucose oxidase (GOx), poly(ethylenimine) (PEI) and carbon nanotube (CNT) is considered, while three cathodic catalysts consist of glutaraldehyde (GA), laccase (Lac), PEI and CNT that are stacked together in different ways. Catalytic activities of the catalysts for glucose oxidation and oxygen reduction reactions (GOR and ORR) are evaluated. As a result, it is confirmed that the catalysts work well for promotion of GOR and ORR. In EBC tests, performances of EBCs including 150 μm-thick membrane are measured as references, while those of membraneless EBCs are measured depending on parameters like glucose flow rate, glucose concentration, distance between two electrodes and electrolyte pH. With the measurements, how the parameters affect EBC performance and their optimal conditions are determined. Based on that, best maximum power density (MPD) of membraneless EBC is 102 ± 5.1 μW · cm^−2^ with values of 0.5 cc · min^−1^ (glucose flow rate), 40 mM (glucose concentration), 1 mm (distance between electrodes) and pH 3. When membrane and membraneless EBCs are compared, MPD of the membraneless EBC that is run at the similar operating condition to EBC including membrane is speculated as about 134 μW · cm^−2^.

Enzymatic Biofuel cells (EBC) are device that converts chemical energy into electrical energy using (i) enzyme as biocatalysts and (ii) glucose/alcohol as biofuels since it was reported in 1964^1^,^2^. The EBC has unique advantages like low-temperature operation, neutral pH, lower maintenance and operation cost and selective catalytic activity. Furthermore, the EBC may well use glucose and oxygen included in human body as its fuel sources^1^,^3^,^4^. In spite of that, it has still several issues to address such as low electrical performance, short long-term stability, use of still expensive membrane and large fuel cell size for embedding inside human body. Enzyme deactivation and denaturation have been already reported as evidences of the drawbacks related to performance and stability of EBC while it is probably that membrane placed in between two electrodes is a main reason for cost increase and size enlargement of the fuel cell system. Such potentially possible problems can be solved by enhancing enzyme immobilization, discovering more appropriate enzyme catalysts and removing the membrane in fuel cell system^4^,^5^,^6^,^7^,^8^,^9^.

Regarding the enzyme immobilization strategy, although physical adsorptions like physical entrapment and chemical bonding like covalent coupling and enzyme cross-linking were attempted, using Layer-by-Layer (LbL) has been recently emerged. According to the LbL structure, (i) multiple repetitive layers are stack together by electrostatic interaction between oppositely charged species and (ii) the stack layers are formed on the nano-sized supporters like carbon nanotube (CNT)^2^,^10^,^11^,^12^,^13^.

As the possible enzyme catalysts, glucose oxidase (GOx) catalyzing glucose oxidation and laccase (Lac) catalyzing oxygen reduction have been considered^14^,^15^. In case of GOx, its relatively large electromotive force and strong compatibility with human body are main advantages while in terms of laccase, its capability catalyzing four electron reduction reactions from O_2_ to H_2_O without production of intermediate H_2_O_2_ can be considered the merit^16^.

To fabricate the viable enzyme-included LbL catalysts, using proper conducting polymer (CP) and/or cross-linker can be affordable option. In this study, poly(ethylenimine) (PEI), glutaraldehyde (GA) and CNT were used as the CP^17^,^18^, cross-linker and supporter, respectively. Regarding PEI, it is charged positively, whereas enzyme molecules and CNT are negatively charged. Such an opposite polarity makes bonding among them strong by electrostatic interaction^19^,^20^. As for cross-linker, GA induces polymerization amid GA and LbL consisting of enzyme/CP/supporter by cross-linking reaction (aldol condensation reaction). As a result, strong covalent bonds (C=N bonds) are formed in between them^21^.

Regarding cost and size of EBC system, optimizing role of membrane included in the EBC may be critical. For doing that, we evaluate two things; first, inspecting optimal membrane thickness and second, removing membrane although the latter is likely to be more effective because the removal of membrane can reduce the size and cost of EBC in a more straightforward way. The membrane usually plays two different roles as separator (i) to alleviate occurrence of mixed potential and (ii) to increase ohmic resistance so that it should be designed (i) what is appropriate membrane thickness if the membrane is utilized and (ii) how to modulate associated parameters if membrane is not used.

In this study, we fabricate EBC system using (i) anode consisting of GA/[[GOx/PEI]_2_/CNT] biocatalyst that is an enzyme structure based on our previous research for glucose oxidation reaction (GOR) and (ii) cathode consisting of three different biocatalysts including GA, Lac, PEI and CNT for oxygen reduction reaction (ORR)^22^. Schematic illustrations indicating enzyme structures of anode and cathode are represented in Fig. 1. Using the enzyme structures, effect of Nafion 117 membrane on EBC performance is initially evaluated and then performances of mediatorless/membraneless EBCs relying on four important parameters are overhauled with quantification of EBC performance by using electrochemical characterizations such as polarization curves and electrochemical impedance spectroscopy (EIS). For measuring them, new EBC kit is designed and Fig. 2 presents photo images and schematic illustrations of the new EBC kit. With that, we anticipate that our study will contribute to establish baseline protocols of mediatorless/membraneless EBC system.

## Results

### Characterizations of the catalysts

Figures S1a–d represent the CV curves showing catalytic activities of (i) GA/[[GOx/PEI]_2_/CNT] used as a catalyst for anode and (ii) Lac-based three different catalysts (Lac/CNT, Lac/PEI/Lac/CNT, and GA/[Lac/PEI/Lac/CNT]) used as catalysts for cathode. Regarding the activity of anodic catalyst, GOR occurring at flavin adenine dinucleotide (FAD) that was deep inside GOx molecules was investigated by CV measurements in N_2_-state and air-state (with O_2_) (Fig. S1a). For the tests, glucose was provided in ranges of 0.1 to 2 mM.

There are two observations about the CV measurements. First, according to the Fig. S1a, in air state, FAD redox peak was downward shifted compared to that measured in N_2_-state. It is due to occurrences of reduction reaction of O_2_ (O_2_^+^2H^+^ + 2e^−^ → H_2_O_2_, increase in cathodic current) and redox reaction of FAD (GOx (FAD) ^+^ 2H^+^ + 2e^−^ ↔ GOx (FADH_2_), existence of redox reaction peaks appeared at −0.469 V vs. Ag/AgCl)^23^. Second, as glucose concentration increased, the CV curve was upshifted, implying that reduction reaction of O_2_ decreased with decrease in cathodic current. In this case, O_2_ was consumed for redox reaction of FAD (GOx (FADH_2_) + O_2_ → GOx (FAD) + H_2_O_2_), not for reduction reaction of O_2_, producing cathodic current. Due to the change in role of O_2_, CV curve downshifted before supply of glucose was upshifted with increase in glucose concentration^22^,^24^,^25^.

**Figure 2:**
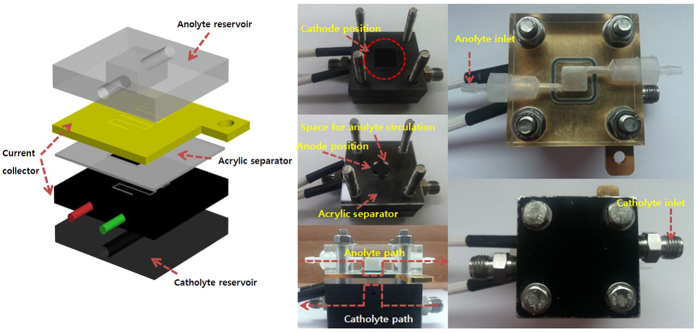
A schematic diagram showing (**a**) individual components of membraneless EBC kit and photo images showing (**b**) catholyte reservoir and cathode electrode, (**c**) anolyte reservoir and anode electrode, (**d**–**f**) cross-sectional, top and bottom view of membraneless EBC kit.

In contrast, regarding the activity of cathodic catalyst, ORR occurring at cupric ions within Lac molecules of the three different Lac-based catalysts was also measured. For comparison, the reaction occurring at N_2_-state (without O_2_) was also measured (Figs S1b–d). In the N_2_-state, redox reaction peaks of cupric ions within Lac molecules of the Lac-based three different catalysts were observed at 0.272, 0.274, and 0.282 V vs. Ag/AgCl. The small reaction peaks were included in inset of the Figs 2b–d, meaning that active sites for ORR of Lac-based catalysts worked well. When O_2_ was supplied, current densities of the three catalysts were down-shifted from 0.1 V vs. Ag/AgCl, while redox peak of cupric ions within Lac molecules was shifted to positive direction and appeared around 0.6 V vs. Ag/AgCl. These observations indicate that ORR of the catalysts takes place appropriately^26^.

To investigate and compare reaction rate of the catalysts, their electron transfer rate constants (K_s_s) and apparent Michaelis-Menten constants (K_m_s) were measured using Laviron’s formula and Michaelis-Menten plot, respectively (Figs S1 and S2)^22^,^27^,^28^. According to the results (Figs S2a–d), K_s_ of GA/[[GOx/PEI]_2_/CNT] catalyst was 12.7 s^−1^, while that of GA/[Lac/PEI/Lac/CNT] catalyst was 8.6 s^−1^, (K_s_s of Lac/CNT and Lac/PEI/Lac/CNT were 6.5 and 8.4 s^−1^).

Even in comparison of K_m_s, K_m_ of GA/[[GOx/PEI]_2_/CNT] catalyst was 0.3 mM, while that of GA/[Lac/PEI/Lac/CNT] catalyst was 0.6 mM (K_m_s of Lac/CNT and Lac/PEI/Lac/CNT were 1.6 and 2.4 mM) (Figs S3a,b). There are two noticeable things for Fig. S3. First, these results about catalytic activities of catalysts prepared for ORR and GOR were well matched with other reference papers^22^,^29^. In addition, K_s_ of the catalysts was higher than that of other similar catalysts, meaning that the catalysts played their role well and second, we swept the entire potential ranges using very slow potential scan rate (5 mV·s^−1^)^22^,^30^. Due to the reason, absolute current density values were low.

**Figure 3:**
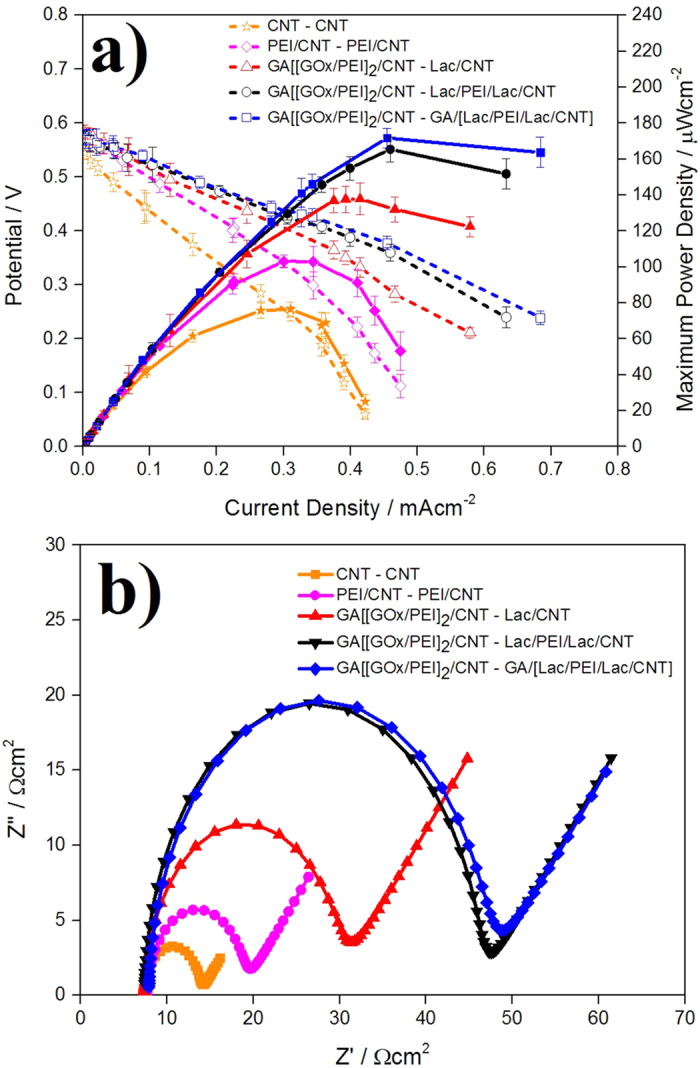
(**a**) Polarization curves of EBCs (i) adopting GA/[ GOx/PEI]_2_/CNT] as anodic catalyst and Lac/CNT, Lac/PEI/Lac/CNT, GA/[Lac/PEI/Lac/CNT] as cathodic catalysts and (ii) adopting catalysts (CNT and PEI/CNT) without enzyme molecules (**b**) Nyquist plots of EBCs adopting the catalyst combinations of (**a**). For the tests, Nafion 117 was uses as membrane and 0.04 M glucose solution (pH 7.4) was provided at a rate of 100 mL · min^−1^ for anode, while 100 cc · min^−1^ O_2_ gas was supplied for cathode. For gaining the corresponding polarization curve, three samples were tested.

To evaluate performances of EBCs including membrane between anode and cathode electrodes, polarization curves of the EBCs adopting GA/[[GOx/PEI]_2_/CNT] and the three different Lac-based structures were measured. Also, to inspect effects of enzyme catalysts on EBC performance, those of EBCs adopting catalysts fabricated without enzyme molecules (CNT and PEI/CNT catalysts) were measured (Fig. 3a). For the tests, 150 μm-thick Nafion 117 membrane was considered, while 0.04 M glucose solution (pH 7.4) was supplied at a rate of 100 mL·min^−1^ (anode) and 100 cc · min^−1^ O_2_ gas was provided (cathode). There were two things to mention about the results. First, regarding EBC performance, maximum power density (MPD) of EBC adopting GA/[[GOx/PEI]_2_/CNT] and GA/[Lac/PEI/Lac/CNT] catalysts was highest (MPDs of EBCs adopting Lac/CNT, Lac/PEI/Lac/CNT and GA/[Lac/PEI/Lac/CNT] catalysts were 138 ± 8.8, 165 ± 7 and 171 ± 5.5 μW · cm^−2^, respectively). It indicates that GA plays a critical role in immobilizing more Lac molecules and activating ORR, followed by improving EBC performance. Second, based on Fig. 3a, because MPDs of EBCs increased 1.4~2.2 fold with adoption of the enzyme molecules (MPD of EBC adopting CNT and PEI/CNT catalysts without enzyme molecules were 78 ± 4 and 100 ± 3.5 μW · cm^−2^ respectively), it was proved that both enzymes (GOx and Lac molecules) were well acted as catalysts for EBC.

To further investigate effect of enzyme catalysts on charge transfer of EBC, charge transfer resistances (R_ct_s) of the EBCs used in Fig. 3a were measured using EIS and Fig. 3b represents their Nyquist plots. In these EIS measurements, for mainly evaluating cathodic catalyst effect, anode was standardized by H_2_ gas. According to the Nyquist plots, when enzyme catalysts were included, R_ct_ was highest when GA/[Lac/PEI/Lac/CNT] was employed in EBC, while the value was lowest when Lac/CNT was employed (R_ct_s of EBCs adopting Lac/CNT, Lac/PEI/Lac/CNT and GA/[Lac/PEI/Lac/CNT] catalysts were 22, 38, and 38 Ω · cm^2^, respectively). On the other hand, when enzyme catalysts were not included, R_ct_ of the EBC was very low (R_ct_ of EBC adopting CNT and PEI/CNT catalysts were 7 and 12 Ω · cm^2^ respectively).

These results make sense. As reported previously, R_ct_ is proportional to loading amount of enzyme molecules (Lac molecules) because the Lac molecule is assemble of proteins that have non-conductive property^31^. Therefore, the R_ct_ trend of Fig. 3b presents that loading amount of Lac in GA/[Lac/PEI/Lac/CNT] is highest, while that of Lac/CNT is lowest. This result is compatible with the polarization curve data of Fig. 3a. It deserves to note that x-axis intercepts of the Nyquist plots are similar together. It demonstrates that membrane resistances (R_s_s) of EBCs are not affected by catalyst types (R_s_s of EBCs adopting catalysts were 7.3~7.9 Ω · cm^2^).

It is also important to investigate whether our enzyme catalysts can work well for a long time. To evaluate the long term stability of enzyme catalysts, catalytic activities of the enzyme catalysts were measured every week for four weeks and the results are represented in Fig. S4a (GA/[[GOx/PEI]_2_/CNT] catalyst for anode) and Fig. S4b (Lac/CNT, Lac/PEI/Lac/CNT and GA/[Lac/PEI/Lac/CNT] catalysts for cathode). For the Fig. S4a, FAD redox reaction peak of GA/[[GOx/PEI]_2_/CNT] catalyst was measured, while cupric ion redox reaction peaks of Lac-based catalysts were measured for the Fig. S4b.

According to the Figs S4a,b, the peak current density of GA/[[GOx/PEI]_2_/CNT] catalyst was maintained to 87% of initial value, while those of Lac-based catalysts were maintained to 89, 75 and 64% of initial value. It can be explained that (i) long term stabilities of all the catalysts are better than that of other similar catalysts, implying that EBCs adopting these catalysts may also keep their stabilities^22^,^32^ and (ii) long term stability of the GA/[Lac/PEI/Lac/CNT] catalyst is best, anticipating that EBC adopting this catalyst will be mostly stable. Taken together, performance and stability measurements of EBCs including membrane between anode and cathode electrodes show that EBC adopting GA/[Lac/PEI/Lac/CNT] catalyst sheds light on promising result.

## Discussion

The results mentioned above discernibly mean that enzyme structures suggested for both anode and cathode electrodes play their role as catalyst. It is now time to determine how performances of membraneless EBCs adopting the enzyme catalysts are affected by four main parameters like glucose flow rate, glucose concentration, interval between two electrodes and electrolyte pH. For doing that, polarization curves of the corresponding EBCs were mainly measured.

**Figure 4:**
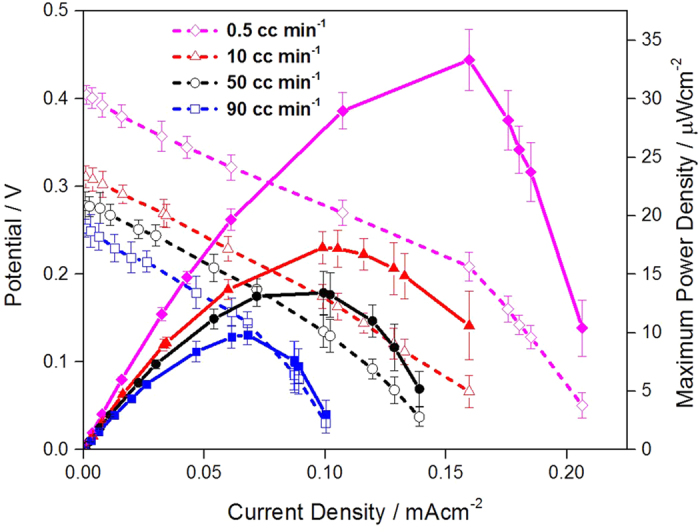
Polarization curves of EBCs adopting GA/[[GOx/PEI]_2_/CNT] as anodic catalyst and GA/[Lac/PEI/Lac/CNT] as cathodic catalyst. For the test, in anode, 0.04 M glucose solution (pH 7.4) was provided at a rate of 0.5, 10, 50 and 90 cc · min^−1^, while for cathode, 100 cc · min^−1^ O_2_ gas was supplied. For gaining the corresponding polarization curve, three samples were tested.

Regarding the glucose flow rate effect, according to polarization curves (Fig. 4), as glucose flow rate increased, open circuit potential (OCP) and MPD of the corresponding EBCs dropped, while the difference in current density region determined by electrolyte resistance and mass transfer was not significant. It is probably attributed to mixed potential occurring in cathode of EBC. Namely, when glucose flow rate is low, glucose and O_2_ fuels are properly acted for GOR and ORR, inducing superior OCP and MPD. However, as glucose flow rate increases, the fuels become easy to access to the opposite electrode, lowering ORR potential and producing self-discharge current by occurrence of mixed potential. Such decreased ORR potential induces decrease in OCP, followed by decrease in MPD of membraneless EBC. More specifically, when glucose flow rate was 0.5 cc · min^−1^, MPD of EBC was 34 ± 2.6 μW · cm^−2^, while the MPD was plummeted to 10 ± 0.9 μW · cm^−2^ with the glucose flow rate of 90 cc · min^−1^. This result was well matched with one reported by Jayashree *et al*., who studied effect of fuel flowrate on performance of microfluidic membraneless fuel cell^33^. Regarding fuel cell using membrane, Zhang *et al*., who investigated effect of fuel flowrate on performance of proton exchange membrane fuel cell (PEMFC) previously reported and their result was also compatible with ours^34^.

**Figure 5:**
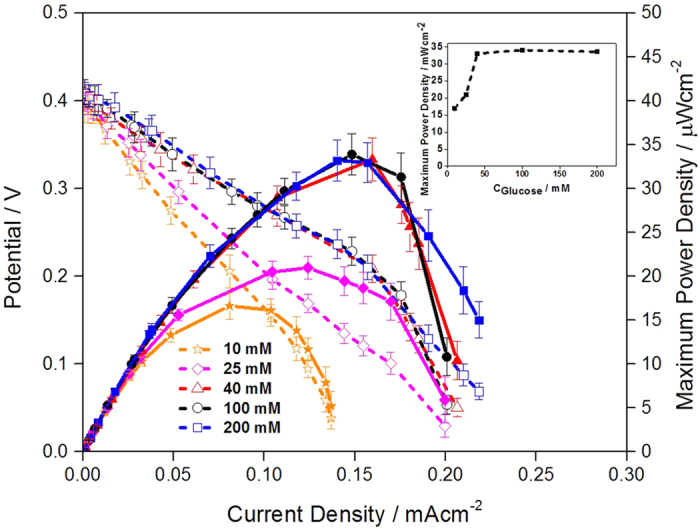
Polarization curves of EBCs adopting GA/[[GOx/PEI]_2_/CNT] as anodic catalyst and GA/[Lac/PEI/Lac/CNT] as cathodic catalyst. Inset indicates a correlation between glucose concentration and MPD of EBCs. For the test, in anode, 10, 25, 40, 100 and 200 mM glucose solution (pH 7.4) was supplied, while for cathode, 100 cc · min^−1^ O_2_ gas was supplied. For gaining the corresponding polarization curve, three samples were tested.

As another parameter affecting performance of membraneless EBC, glucose concentration was regarded and its effect on membraneless EBC performance was evaluated. For the EBC tests, glucose concentrations of 10, 25, 40, 100 and 200 mM (pH 7.4) were provided with glucose flow rate of 0.5 cc · min^−1^. Their polarization curves are presented in Fig. 5. According to that, as glucose concentration increased up to 40 mM, MPD also increased, whereas there was no magnificent change in MPD when glucose concentration was higher than 40 mM. It means that in 10~40 mM range, GOR is promoted, producing increase in MPD of EBC. In contrast, in 40~200 mM range, GOR is saturated and the MPDs remain unchanged. Inset of the Fig. 5 indicates a correlation between MPD of EBCs and glucose concentrations.

In detailed evaluations, it is clear that under different glucose concentrations, performances of EBCs measured by polarization curves mainly depend on the difference in current density region occurred by electrolyte resistance and mass transfer with little change in OCP of the EBCs (see and compare the current-potential lines of Fig. 5). It is explained that in 10~40 mM range, increase in glucose concentration promotes mass transfer by increase in glucose concentration gradient and such facilitated mass transfer reduces performance loss caused by mass transfer, producing enhancement in EBC performance. However, in 40~200 mM range, glucose concentration gradient and their mass transfer are saturated and performances of EBCs are maintained without further alteration^35^. Our result was similar with fuel cell using membrane that reported by Assumpção *et al*., who investigated effect of ethanol concentration on performance of direct ethanol fuel cell (DEFC)^36^. According to their result, when membrane was used, change in ethanol concentration (liquid, anode) was more dominant factor to determine the DEFC performance than change in O_2_ concentration (gas, cathode).

**Figure 6:**
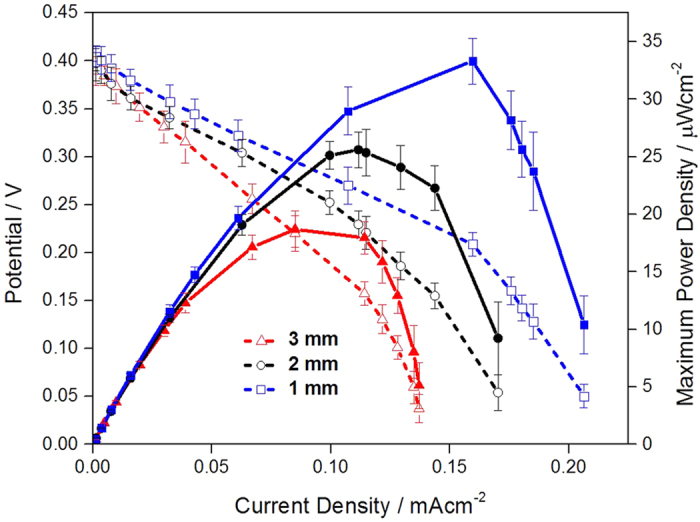
Polarization curves of EBCs adopting GA/[[GOx/PEI]_2_/CNT] as anodic catalyst and GA/[Lac/PEI/Lac/CNT] as cathodic catalyst. For the test, in anode, 0.04 M glucose solution (pH 7.4) was provided at a rate of 0.5 cc · min^−1^, while for cathode, 100 cc · min^−1^ O_2_ gas was supplied. As distance of separator placed between anode and cathode, 1, 2 and 3 mm were assigned. For gaining the corresponding polarization curve, three samples were tested.

Effect of distance between anode and cathode on performances of membraneless EBCs was also critical and related polarization curves were measured (Fig. 6). For the EBC tests, three different distances of 1, 2 and 3 mm were assigned with glucose flow rates of 0.5 cc·min^−1^ and glucose concentration of 40 mM (pH 7.4). According to the Fig. 5, there are two noticeable things. First, as the distance between electrodes was reduced, MPDs increased. Such an inverse proportion between distance and EBC performance is attributed to ohmic resistance loss. The ohmic resistance has the following relationship; R = ρ (L/A), here, R is ohmic resistance, ρ is resistivity of electrolyte, L is distance between electrodes and A is corresponding area of each electrode. As the distance between electrodes increased, ohmic resistance increased and with that, potentials at current density region affected by the ohmic resistance were dropped and performance of EBC also decreased.

Second, as a distance between two electrodes was reduced, MPDs of the EBCs increased in a linear progression (when the distance is 3, 2, and 1 mm, MPDs of the EBCs is 19 ± 1.6, 26 ± 1.5 and 33 ± 2.6 μW · cm^−2^). The linear relationship among ohmic resistance, distance between two electrodes and MPD of the EBC is explained that the ohmic resistance loss is probably one of dominant factors to determine MPD of membraneless EBC because MPDs of membraneless EBCs follow up trend of ohmic resistance loss in all three different distances of 1, 2 and 3 mm.

Such results are reasonable and compatible with previously reported ones^37^. In particular, according to the result performed by Liu *et al*., who did research on performance of direct methanol fuel cell (DMFC) using different MEA thickness, as distance between electrodes in DMFC decreased (decrease the membrane thickness), its performance increased due to reduction of ohmic resistance between electrodes^38^.

**Figure 7:**
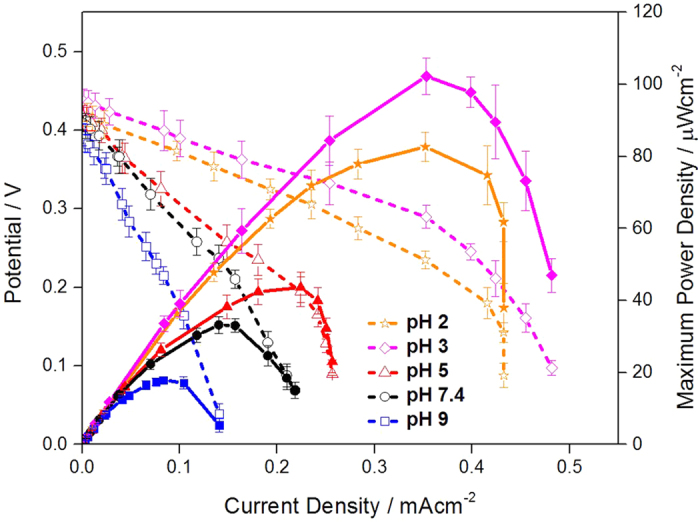
Polarization curves of EBCs adopting GA/[[GOx/PEI]_2_/CNT] as anodic catalyst and GA/[Lac/PEI/Lac/CNT] as cathodic catalyst. For the test, glucose solutions showing five different pHs like 2, 3, 5, 7.4 and 9 were adopted with distance of 1 mm, glucose flow rates of 0.5 cc · min^−1^ and glucose concentration of 40 mM. For cathode, 100 cc · min^−1^ O_2_ gas was supplied. For gaining the corresponding polarization curve, three samples were tested.

Effect of pH of electrolyte (glucose solution) on performance of membraneless EBCs was also investigated because the pH of glucose solution filling empty acrylic separator placed between two electrodes affected the EBC performance. For the EBC tests, glucose solutions showing five different pHs like 2, 3, 5, 7.4 and 9 were adopted with distance of 1 mm, glucose flow rates of 0.5 cc · min^−1^ and glucose concentration of 40 mM. Their polarization curves are represented in Fig. 7. According to the Fig. 7, when pH of glucose solution was 3~5, performance of EBC was best (MPD of the EBC was 102 ± 5.1 μW · cm^−2^). Generally, GOx (anodic catalyst) was mostly activated near to pH 7~8, while activity of Lac (cathodic catalyst) was best near to pH 3~5. Thus, it means that the electrolyte pH effect is mostly dominated by catalytic activity of Lac molecules, confirming that performance of this membraneless EBC depends on cathodic reaction.

This pH dependence on performance of membraneless EBC may be ascribed to change of hydroxide anion (OH^−^) controlled by electrolyte pH. When the pH is higher than 5, OH^−^ ions also increase with activated reduction reaction. Such increased OH^−^ ions then make activity of Lac molecules weak because the OH^−^ ions are stuck to the active sites of Lac molecules, preventing O_2_ molecules from reacting at the active sites^39^. Also, this result showing the best performance in pH 3 is related to effect of electrolyte pH on activity of GOx immobilized on CNT, implying that the activity is not obviously lowered even under acidic electrolyte condition. It indicates that GOx molecules are protected well in our catalytic structure and work for anodic reaction without getting fully deactivated. This result was also matched with that reported by Liu *et al*. and Tan *et al*.^40^,^41^.

It is noticeable that the best MPD of the EBC (102 ± 5.1 μW · cm^−2^) is also pretty excellent value compared to that of other membraneless EBCs. For instance, MPD of membraneless EBC consisting of GOx/PPy/CNT (anode) and Lac/PPy/CNT (cathode) was 30 μW · cm^−2^ 42. That consisting of GDH/Poly-lysine (anode) and PDMS/Pt (cathode) was 32 μW · cm^−2^ 43. That consisting of GOx/Au electrode (anode) and Cytrochrome oxidase/Au electrode (cathode) was 21 μW · cm^−2^ 44. Also, that consisting of GOx/C (anode) and MvBOD at pH 7.4 (cathode) was 43 μW · cm^−2^ 45.

**Figure 8:**
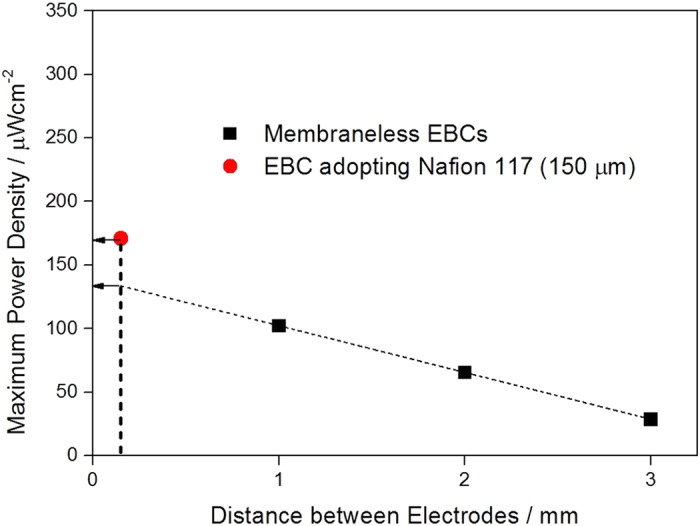
A linear regression between MPDs of membraneless EBCs and distance between electrodes and comparison of MPDs between membraneless EBC and EBC adopting Nafion 117.

As we already explained, EBCs including membrane and membraneless EBCs showed their own performances. However, since there is a difference in the distance between two electrodes (in EBCs including membrane, the distance between two electrodes is 150 μm, while the minimum distance between two electrodes is 1 mm in membraneless EBCs), it is not possible to compare performances of the two EBCs in the status quo. To compare them in a proper manner, we estimated the regression curve between MPDs of the membraneless EBCs gained under the condition of 0.04 M glucose solution (pH 3) and distance between two electrodes (Fig. 8). Based on the Fig. 8, the linear progression calculated was MPD = −0.037 mm + 0.14. With the correlation, MPD of the membraneless EBC when the distance between two electrodes reaches 150 μm is extrapolated and the value is speculated as 134 μW · cm^−2^. Although the MPD is still less than that of EBC including membrane (171 ± 5.5 μW · cm^−2^), it is obviously viable result.

Stability of membraneless EBC was also measured for four weeks by tracking variance in its MPD. For the tests, optimal conditions of the aforementioned parameters were used and three samples were experimented for determining MPD. According to the MPD results represented in Fig. S5, MPD was maintained up to 77% even after 4 weeks (from 102 ± 5.1 to 79 ± 3.5 μW · cm^−2^). Although further efforts are required to improve the stability, it is reasonable to say that our membraneless EBC system is relatively stable.

In this study, performances of mediatorless/membraneless EBCs adopting enzyme catalysts and catalytic activities of the enzyme catalysts were investigated. When the catalytic activities of catalysts for GOR and ORR were evaluated, they showed reasonable reaction trend with excellent activity values. In EBC tests, performances of EBCs adopting Nafion 117 membrane were evaluated. According to the EBC tests, performance of EBC adopting GA/[Lac/PEI/Lac/CNT] as cathodic catalyst was best (171 ± 5.5 μW · cm^−2^), meaning that (i) GA promoted immobilization of more Lac molecules, followed by activation of the ORR and (ii) cathodic reaction (ORR) was a rate determining step to affect performance of EBC.

When performances of membraneless EBCs were measured, four different parameters like glucose flow rate, glucose concentration, interval between two electrodes and electrolyte pH were considered. In the polarization curve measurements, how the parameters affected the performances of EBCs was clarified and the best condition of the parameters was obtained. As a result of that, MPD of membraneless EBC was reached to 102 ± 5.1 μW · cm^−2^ and as the optimal conditions, 0.5 cc · min^−1^ (glucose flow rate), 40 mM (glucose concentration), 1 mm (interval between two electrodes) and pH 3 were determined.

To compare performances of EBCs including membrane and membraneless EBC in equivalent state, MPD of the membraneless EBC was expressed as distance between electrodes. With that, a linear progression of MPD = −0.037 mm + 0.14 was obtained and the MPD value of 134 μW · cm^−2^ was calculated by the extrapolation equation. Although this value is still lower than that of EBC including membrane, it is clearly promising result.

## Methods

### Materials

Multiwall carbon nanotubes (MWCNT) (its purity is higher than 90%) were obtained from NanoLab (Brington, MA). Glucose oxidase (GOx, from Aspergillus niger type X-S, 150.000 U · g^−1^ solid), Laccase (from Trametes versicolor, 0.92 U·mg^−1^ solid), glutaraldehyde (GA) solution, and polyethylenimine (PEI, 50% solution) were purchased from Sigma Aldrich (Milwaukee, WI, USA). Sodium-acetate buffer pH 5 was made from mixture between sodium acetate and acetic acid and that was purchased from Sigma Aldrich (Milwaukee, WI, USA).

### Fabrication of anodic and cathodic catalysts

GA/[[GOx/PEI]_2_/CNT] was prepared by LbL deposition between GOx molecules, PEI, and CNT. By carrying out preliminary optimization process, it was confirmed that 5 mg·mL^−1^ CNT, 2.5 mg·mL^−1^ PEI and 4 mg·mL^−1^ GOx were optimal conditions for synthesizing optimal [GOx/PEI]_2_ layer. In detailed explanation, 5 mg·mL^−1^ CNT was mixed with 2.5 mg·mL^−1^ PEI solution. Supernatant of the mixture was then removed and washed with DI water to remove excess PEI. 4 mg·mL^−1^ GOx solution was then added to the mixture. This process was repeated twice to fabricate [GOx/PEI]_2_ layer. The final mixture was mingled with 0.5 w/v % GA solution. The GA included mixture was then centrifuged at 14,000 rpm for 10 min. The excess GA was removed by using DI water 2 times to avoid further cross-linking process.

Three different types of catalysts including Lac molecules were prepared for cathode. The catalysts are denoted as Lac/CNT, Lac/PEI/Lac/CNT, and GA/[Lac/PEI/Lac/CNT], respectively. Same to anodic catalyst, by carrying out preliminary optimization process, it was confirmed that 0.4 mg·mL^−1^ Lac, 2.5 mg·mL^−1^ PEI and 0.5 w/v % GA were optimal conditions for synthesizing optimal cathodic catalysts. In detailed explanation, Lac/CNT catalyst was prepared by LbL deposition between Lac molecules and CNT. First, 10 mg CNT and 10 mL of 0.1 M sodium-acetate buffer (pH 5) were mixed. The mixture was further dissolved into 0.4 mg·mL^−1^ Lac solution under 0.1 M sodium-acetate buffer (pH 5). The mixture was then incubated for 2.5 h and centrifuged at 13,000 for 10 min. Lac/PEI/Lac/CNT catalyst was prepared by additional process of the Lac/CNT catalyst. The suspended Lac/CNT catalyst was mixed with 2.5 mg·mL^−1^ PEI solution (in 0.5 M NaCl (pH 5)). Supernatant of the mixture was then removed and washed with DI water to remove excess PEI. 0.4 mg·mL^−1^ Lac solution (in 0.1 M sodium-acetate buffer pH 5) was added to the mixture and the final mixture underwent the same incubation process to Lac/CNT catalyst. GA/[Lac/PEI/Lac/CNT] catalyst was prepared by additional process of the Lac/PEI/Lac/CNT catalyst. The suspended Lac/PEI/Lac/CNT catalyst was mixed with 0.5 w/v % GA solution. The mixture was then centrifuged at 13,000 rpm for 10 min. The excess GA was removed by using DI water 2 times to avoid further cross-linking process. All the catalysts were dipped in sodium-acetate buffer (pH 5) at 4 °C when not in use.

### Catalytic and electrochemical evaluations of laccase-based catalysts

A computer connected potentiostat (Bio-Logic SP-240, USA) was used for electrochemical measurements. For the half-cell tests like CV, Pt wire and Ag/AgCl (soaked in 3.0 M KCl) were served as counter and reference electrodes respectively, while catalysts loaded on glass carbon electrodes (GCE) were acted as working electrode. For loading of catalysts, catalytic powder was mixed with 1 mL of H_2_O and then, 10 μm of catalytic ink was dropped on the GCE. The catalytic ink-loaded working electrode was dried for 45 min. After drying, 5 wt % Nafion solution was coated on the working electrode to complete configuration of working electrode. As for electrolyte, 0.01 M PBS (pH 7.4) was considered.

For measuring polarization curve and membrane/charge transfer resistances (R_s_/R_ct_), new EBC kit was designed with active surface area of 1.0 cm^2^. When membrane was included, the kit was consisted of catholyte reservoir, cathode electrode, membrane, anode electrode and anolyte reservoir. On the other hand, when used as membraneless EBC kit, membrane was removed and instead, acrylic separator was inserted. As the membrane, Nafion 117 (150 μm thick) was chosen.

When membrane was used, to prepare for cathode including enzyme catalyst, the three different catalysts were air-sprayed on a carbon paper (GDL 35BC, CNL Energy, Korea) and placed in cathode part of the EBC kit. After the cathode was ready, it was stuck to the membrane, while GA/[[GOx/PEI]_2_/CNT] catalyst was air-sprayed on the carbon paper and placed in anode part. When membrane was removed, acrylic separator that has 1–3 mm distance was used for EBC tests. After the EBC was fabricated, 0.01~0.2 M glucose solution of feeding rate of 0.5~90 mL · min^−1^ and 100 cc · min^−1^ O_2_ gas were supplied to anolyte and catholyte reservoirs, respectively.

To measure polarization curves from the EBC single cell, Bio-Logic (SP-240, USA) potentiostat was used. It was also connected with a frequency response analyzer (FRA). By coupling FRA with the potentiostat, the power output was determined. The EIS measurements were performed at the open cell potential condition of the EBC. The impedance spectra were decided in the frequency range between 2 MHz and 10 Hz with 10 steps per decade and modulating potential was fixed as 10 mV. To attain the membrane/charge transfer resistances (R_s_/R_ct_) in the corresponding EBC single cells, its Nyquist plot was measured. Nyquist plot produced is available for anode and cathode, indicating that semicircle of the Nyquist plot reflects R_ct_ of the full cell, while intercept of x axis stands for R_s_.

## Additional Information

**How to cite this article**: Christwardana, M. *et al*. Fabrication of Mediatorless/Membraneless Glucose/Oxygen Based Biofuel Cell using Biocatalysts Including Glucose Oxidase and Laccase Enzymes. *Sci. Rep.* **6**, 30128; doi: 10.1038/srep30128 (2016).

## Supplementary Material

Supplementary Information

## Figures and Tables

**Figure 1:**
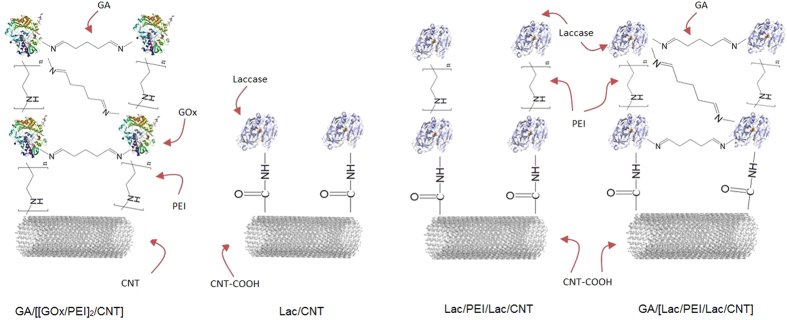
Schematic illustrations showing enzyme structures of GA/[[GOx/PEI]_2_/CNT] as anode while Lac/CNT, Lac/PEI/Lac/CNT, and GA/[Lac/PEI/Lac/CNT as cathode.
