# Supplementary material for: Fabrication of Mediatorless/Membraneless Glucose/Oxygen Based Biofuel Cell using Biocatalysts Including Glucose Oxidase and Laccase Enzymes
Source: Sci Rep. 2016 Jul 18;6:30128. doi: 10.1038/srep30128 (PMC4948020; doi:10.1038/srep30128)
Supplement: Supplementary Information [file srep30128-s1.pdf]

Supporting Information

Fabrication of Mediatorless/Membraneless Glucose/Oxygen Based Biofuel Cell using Biocatalysts Including Glucose Oxidase and Laccase Enzymes

Marcelinus Christwardana, Ki Jae Kim and Yongchai Kwon*

Contents

I. Figures

Figure S1

Figure S2

Figure S3

Figure S4

Figure S5

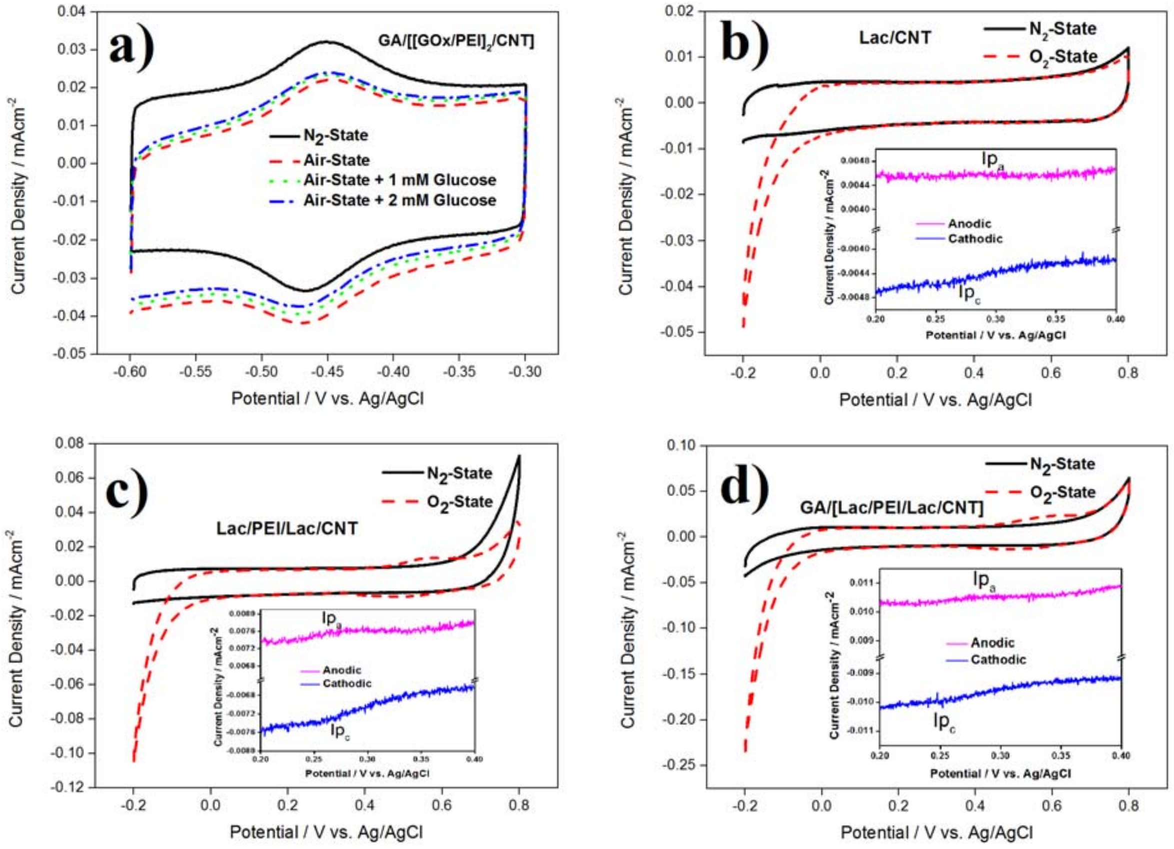

Figure S1. Cyclic voltammograms (CVs) of (a) GA/[[GOx/PEI]$_2$/CNT] operated at N_2-state and air-state. The concentrations of glucose provided at air-state were 0, 1, and 2 mM glucose, (b) Lac/CNT, (c) Lac/PEI/Lac/CNT and (d) GA/[Lac/PEI/Lac/CNT] operated at N_2-state and air-state. Insets of (b)~(d) indicate redox peak of cupric ions within Lac molecule that was shifted to positive direction and appeared around 0.6 V vs. Ag/AgCl at O_2-state. For the CV tests, 0.01 M PBS (pH 7.4) was used as electrolyte and potential scan rate was 5 mV·s^{-1}.

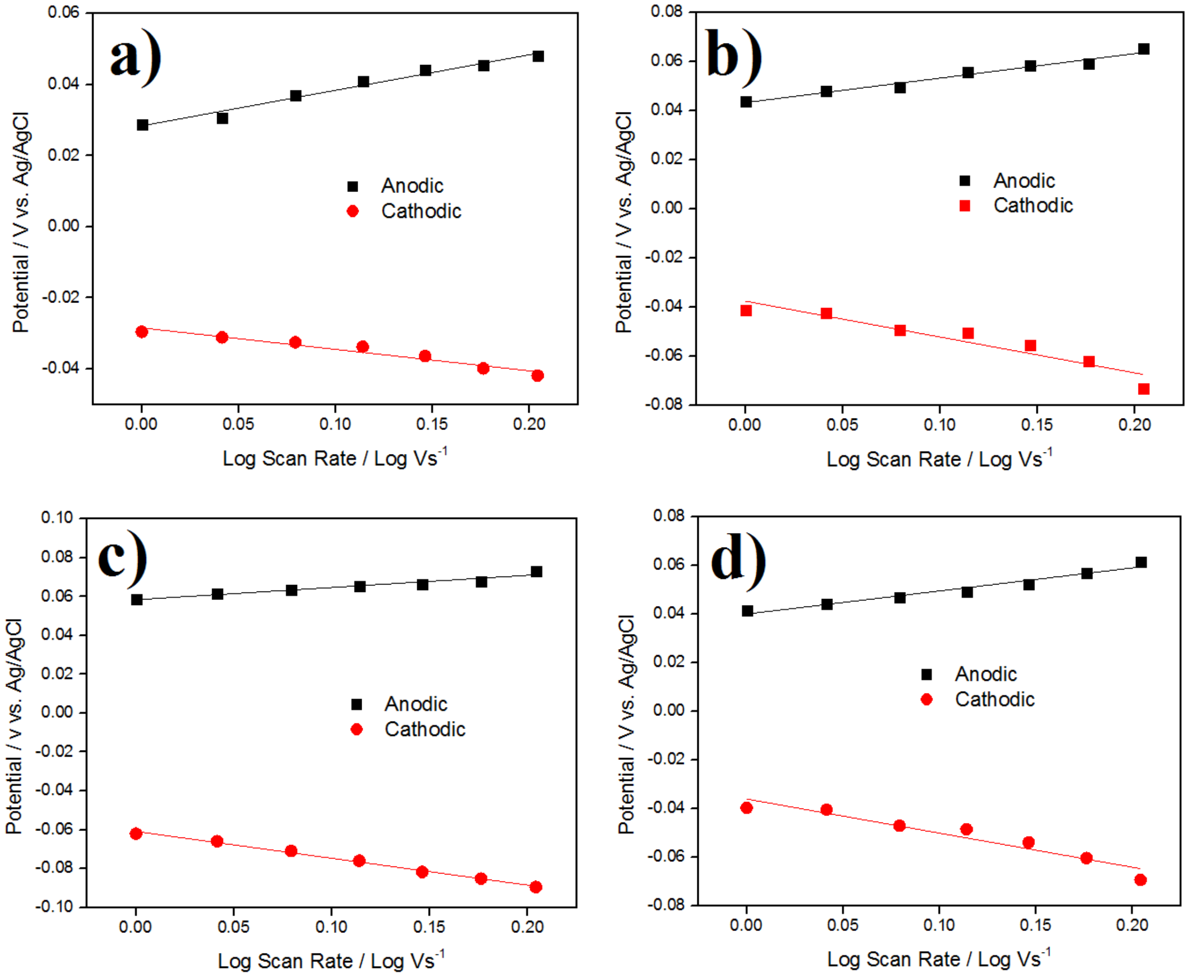

Figure S2. Laviron's plots for (a) GA/[[GOx/PEI]$_2$/CNT] as anode and (b) Lac/CNT, (c) Lac/PEI/Lac/CNT and (d) GA/[Lac/PEI/Lac/CNT] as cathode. For the CV tests, 0.1 M PBS (pH 7.4) was served as electrolyte and potential scan rate was 5 mV·s^{-1}.

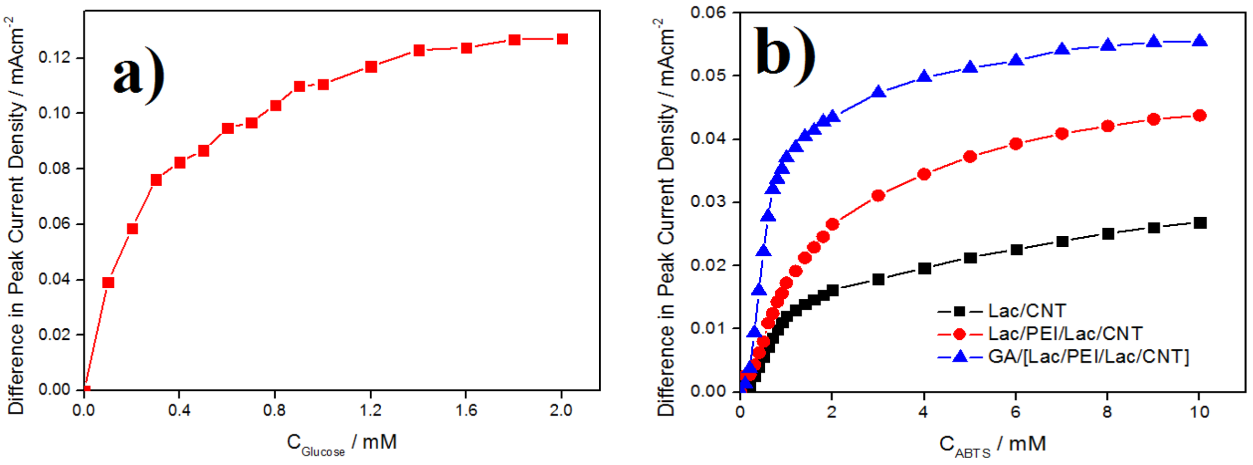

Figure S3. Michaelis-Menten plots (a) GA/[[GOx/PEI]$_2$/CNT] as anode and (b) Lac/CNT, Lac/PEI/Lac/CNT and GA/[Lac/PEI/Lac/CNT] as cathode. In the tests of the anodic catalyst, 0.1 M PBS (pH 7.4) was used as an electrolyte at N_2 state and 0~2 mM glucose was supplied, while In the tests of the cathodic catalysts, 0.1 M PBS (pH 7.4) was used as an electrolyte at N_2 state and 0~10 mM ABTS was supplied. In all the tests, potential scan rate used was 5 mV·s^{-1}.

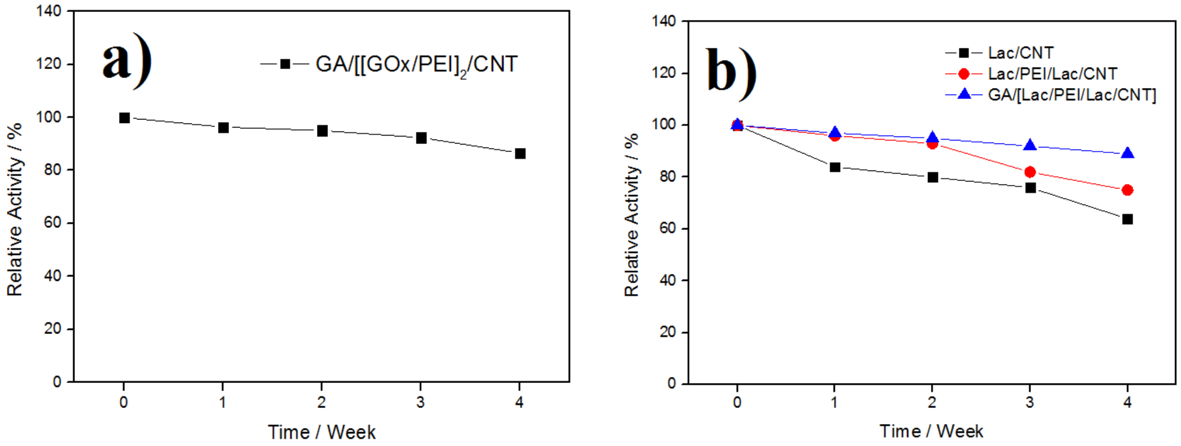

Figure S4. Stability evaluations by measurements of catalytic activities of (a) GA/[GOx/PEI]$_2$/CNT] and (b) Lac/CNT, Lac/PEI/Lac/CNT, GA/[Lac/PEI/Lac/CNT] catalysts for four weeks.

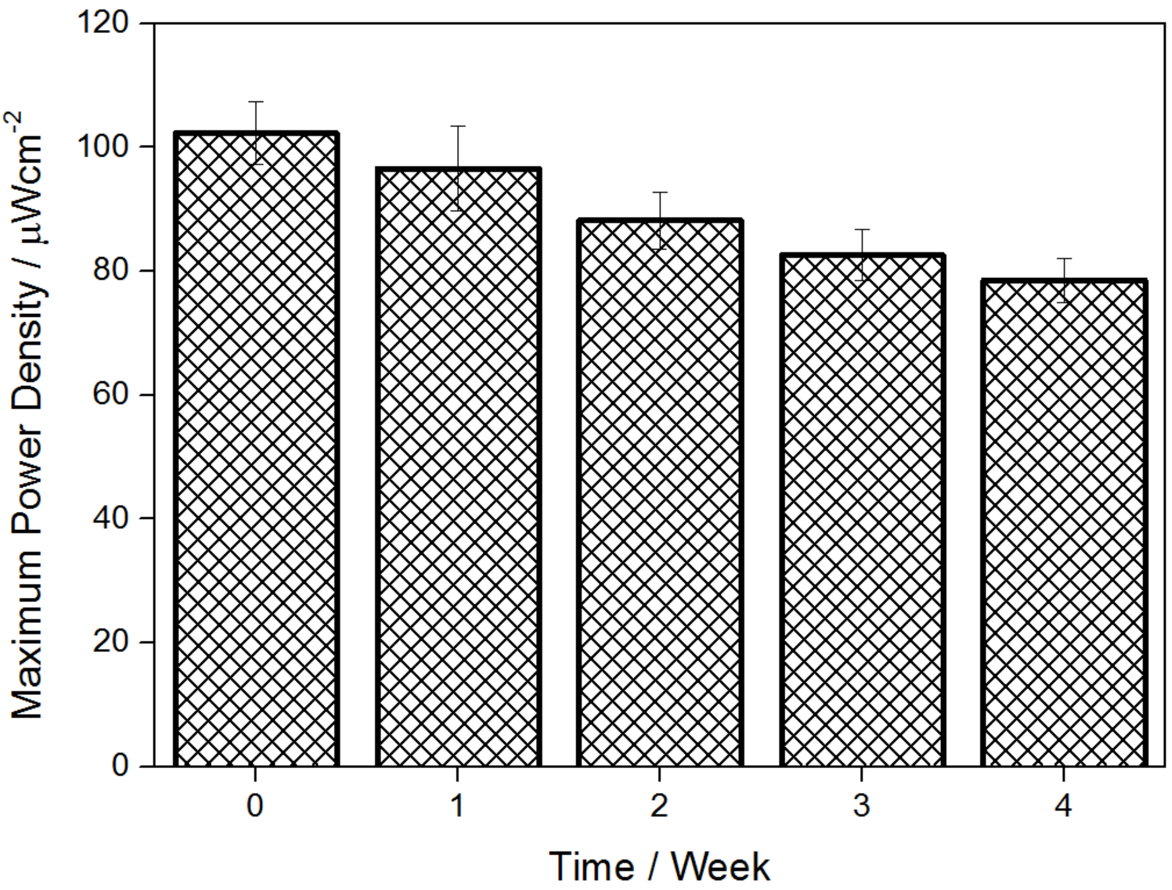

Figure S5. Stability evaluations by measurements of membraneless mediatorless EBC with GA/[GOx/PEI]$_2$/CNT] as anode catalyst and GA/[Lac/PEI/Lac/CNT] as cathode catalysts for four weeks. In this experiment, 0.1 M PBS (pH 3) was used as an electrolyte, 40 mM glucose solution was supplied with flowrate 0.5 cc min^{-1}, and the distance between electrode was 1 mm. For cathode, 100 cc·min^{-1} O_2 gas was supplied.
